# CHK1 inhibition as a strategy for targeting fanconi anemia (FA) DNA repair pathway deficient tumors

**DOI:** 10.1186/1476-4598-8-24

**Published:** 2009-04-16

**Authors:** Clark C Chen, Richard D Kennedy, Samuel Sidi, A Thomas Look, Alan D'Andrea

**Affiliations:** 1 Department of Radiation Oncology, Dana-Farber Cancer Institute, Harvard Medical School, 44 Binney Street, Boston, MA 02115, USA; 2 Department of Neurosurgery, Beth Israel Hospital, Harvard Medical School, 110 Lowry Medical building, Boston, MA 02115, USA; 3 Department of Pediatric Oncology, Dana-Farber Cancer Institute, Harvard Medical School, 44 Binney Street, Boston, MA 02115, USA

## Abstract

**Background:**

DNA repair deficient tumor cells have been shown to accumulate high levels of DNA damage. Consequently, these cells become hyper-dependent on DNA damage response pathways, including the CHK1-kinase-mediated response. These observations suggest that DNA repair deficient tumors should exhibit increased sensitivity to CHK1 inhibition. Here we offer experimental evidence in support of this hypothesis.

**Results:**

Using isogenic pairs of cell lines differing only in the Fanconi Anemia (FA) DNA repair pathway, we showed that FA deficient cell lines were hypersensitive to *CHK1 *silencing by independent siRNAs as well as CHK1 pharmacologic inhibition by Gö6976 and UCN-01. In parallel, an siRNA screen designed to identify gene silencings synthetically lethal with CHK1 inhibition identified genes required for FA pathway function. To confirm these findings *in vivo*, we demonstrated that whole zebrafish embryos, depleted for *FANCD2 *by a morpholino approach, were hypersensitive to Gö6976. Silencing of FA genes led to hyper-activation of CHK1 and vice versa. Furthermore, inactivation of CHK1 in FA deficient cell lines caused increased accumulation of DNA strand and chromosomal breakages. These results suggest that the functions subserved by CHK1 and the FA pathway mutually compensate in maintaining genome integrity. As CHK1 inhibition has been under clinical trial in combination with cisplatin, we showed that the FA specific tumoricidal effect of CHK1 inhibition and cisplatin was synergistic.

**Conclusion:**

Taken together, these results suggest CHK1 inhibition as a strategy for targeting FA deficient tumors.

## Background

Human cancers exhibit genomic instability and heightened drug sensitivity due to underlying defects in DNA repair or cell cycle regulation [1-3]. The specific pathways affected may be predictive of the tumor's drug sensitivity and clinical outcome. For some tumors, loss of one DNA repair pathway may result in hyper-dependence on a second, compensatory DNA repair pathway. Therapeutic gain may be achieved by inhibition of this second pathway.

The Fanconi Anemia pathway (FA) is a DNA repair pathway required for cellular response to DNA cross-linking agents such as mitomycin C (MMC) and cisplatin (CDDP). The thirteen known FA proteins cooperate in this pathway, leading to the monoubiquitination of the FANCD2/FANCI hetero-dimer, activating DNA crosslink repair [4]. Disruption of any of the proteins in the FA pathway, either by germline or somatic mutations, leads to the characteristic cross-linker hypersensitivity and chromosome instability.

Many human cancers have an acquired disruption of the FA pathway. Loss of the pathway has been observed in brain cancer [5], ovarian carcinomas [6], cervical cancer [7], head and neck squamous cell carcinomas [8], and myeloid leukemias [9]. It is estimated that approximately 15% of all tumors harbor defects in the FA pathway [10]. In most cases, disruption results from biallelic methylation and silencing of one of the upstream FA genes, *FANCF*. Disruption of the pathway can also result from loss of *BRCA2/FANCD1 *expression, as observed in breast, ovarian, and pancreatic tumors [10]. FA pathway deficient tumors have recently been shown to be hyper-dependent on a different DNA repair mechanism mediated by the ATM kinase [11].

DNA repair through the FA pathway occurs primarily during S phase of the cell cycle. Accordingly, FA tumor cells acquire extensive DNA damage in S phase. These lesions persist throughout the remainder of the S and G2 phase, ultimately activating the G2/M checkpoint [12,13]. As such, increased accumulation of cells in the G2 phase of the cell cycle is a useful diagnostic feature of FA cells [14]. This accumulation correlates with the hyper-activation of a G2/M checkpoint [15]. We hypothesize that FA cells may be hyper-dependent on this G2/M checkpoint for viability, since the checkpoint activation allows for the repair of damaged DNA prior to mitosis.

The G2/M checkpoint of FA cells is regulated by the checkpoint kinase, CHK1. CHK1 is activated by the ATR kinase in response to DNA damages that stall replication fork progression [16]. Upon activation, CHK1 functions by phosphorylating Cdc25c, thereby halting the transition of cells from G2 to M phase. Several CHK1 inhibitors are currently undergoing clinical trials as anti-neoplastic agents [17,18]. These inhibitors are used largely in combination with other DNA damaging agents including cisplatin [19], fluorouracil [20], topotecan [21], and cytarabine [22].

Given the hyper-dependence of FA cells on the G2/M checkpoint and the critical role of CHK1 in mediating this checkpoint, we hypothesized that FA pathway deficient tumors may be hypersensitive to CHK1 inhibition. Here, we provide both *in vitro *and *in vivo *evidence that FA deficient tumor cells are hypersensitive to inhibition of CHK1, particularly when combined with cisplatin therapy. The functions of these two pathways appear compensatory as inactivation of one leads to the hyper-activation of the other. Taken together, these results suggest that the integrity of the FA pathway represents a critical molecular determinant of therapeutic response to CHK1 inhibition.

## Results

### FA pathway deficient cells are hypersensitive to CHK1 inhibitors

FA cells accumulate in the G2 phase of the cell cycle, and this accumulation is more pronounced after exposure to exogenous DNA damaging agents [12]. The G2 accumulation results, at least in part, from hyperactive CHK1 activity. This CHK1 hyperactivity may serve as a compensatory mechanism for FA pathway deficiency. We reasoned therefore that FA cells may be hypersensitive to CHK1 inhibition.

**Figure 1:**
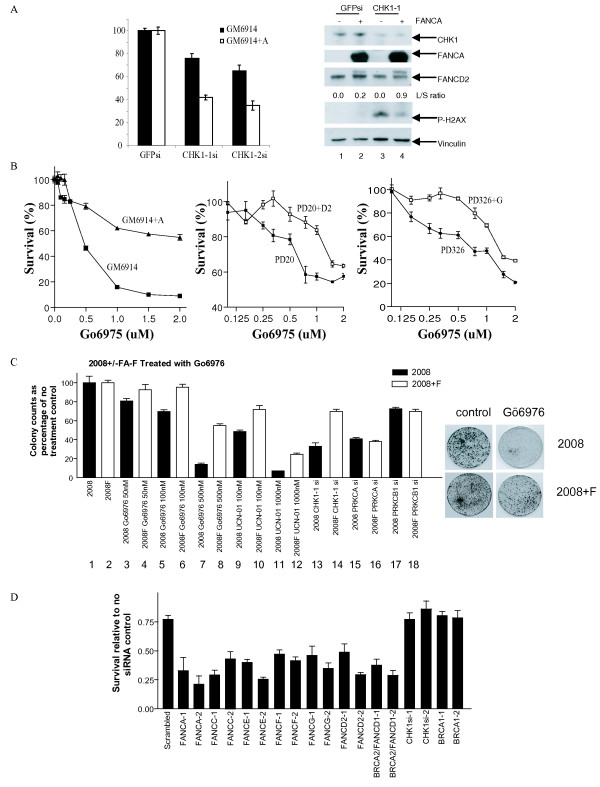
**FA cells are hypersensitive to CHK1 silencing and inhibition**. (A) Response of isogenic FANCA deficient (GM6914) and proficient (GM6914+A) lines to *CHK1 *silencing. Cells were treated with siRNA targeting *GFP *(bars 1 and 2), *CHK1-1 *siRNA (bars 3 and 4) or *CHK1-2 *siRNA (bars 5 and 6). Western blot analysis: GM6914 (lanes 1, 3); GM6914+A (lanes 2, 4). (B) Response of *FANCA *(GM6914), *FANCD2 *(PD20 cells), and *FANCG *(PD326) deficient lines to CHK1 inhibition by Gö6976. Cells were exposed to Gö6976 for 24 hrs and then incubated for 5 days before viability was determined by the Cell Titer-Glo assay (Promega). (C) A FA pathway deficient ovarian cancer cell line is hyper-sensitive to CHK1 inhibition. A 14 day colony count assay comparing the response of *FANCF *deficient 2008 ovarian cancer cells (bars 1, 3, 5, 7, 9, 11, 13, 15, and 17) versus *FANCF *corrected 2008F cells (bars 2, 4, 6, 8, 10, 12, 14, 16, and 18) after treatment with Gö6976, UCN-01, and siRNA against *CHK1*, *PRKCα*, *PRKCβ1*, and *GFP*. Viability of each target siRNA treated cell line was calculated as a percentage of the GFP siRNA treated cells. (D) Disruption of the FA pathway sensitizes cells to Gö6976. HeLa cells were transfected with the various siRNAs for 24 hrs followed by treatment by either Gö6976 (500 nM) or DMSO. Each bar represents the ratio of cellular viability between Gö6976 and DMSO treatment.

To test this hypothesis, we first examined the effect of CHK1 silencing on the clonogenic survival of a FANCA deficient cell line (GM6914) and its isogenic FANCA corrected cell line (GM6914+A). Two distinct siRNAs directed against CHK1 were tested in order to minimize the likelihood of off-target effects (Figure 1A). With both siRNAs, GM6914 (FANCA-deficient) cells were more sensitive to CHK1 knockdown than the corrected cell line. For CHK1-1 siRNA, the uncorrected cells exhibited only 42% viability after siRNA treatment (lane 3), while the corrected cells showed 76% viability (lane 4). For CHK1-2 siRNA, the uncorrected cells exhibited only 40% viability after siRNA treatment (lane 5), whereas the corrected cells showed 65% viability (lane 6). Of note, we previously reported that FA deficient cell lines are hypersensitive to ATM inhibition [11]. The magnitude of the FA specific killing by CHK1 silencing reported here is comparable to that observed for ATM silencing.

A Western blot was performed to confirm silencing of CHK1 and better characterize the molecular nature of the FA-CHK1 interaction (Figure 1A, Western blot). Silencing with the CHK1-1 siRNA resulted in decreased CHK1 protein levels and increased phospho-H2Ax levels, particularly in the FANCA deficient cells (lanes 3, 4). This result suggests that the CHK1 and FA genes function in compensatory manner to maintain genome integrity.

Consistent with this model, the GM6914+FANCA corrected cell line demonstrated enhanced FANCD2 monoubiquitination following knockdown of CHK1 (L/S ratio of 0.9 vs 0.2), suggesting that the FA pathway is activated following loss of CHK1 function (Figure 1B compare lanes 2 and 4 in the Western blot).

### FA deficient cells are hypersensitive to pharmacologic CHK1 inhibition

To further safeguard against siRNA "off-target" effects, we wished to confirm our observation using a pharmacologic inhibitor of CHK1. Recent studies have indicated that some of the small molecular inhibitors initially thought to be CHK1 specific possessed activities against related kinases [23]. As more small molecule kinase inhibitors are subjected to detailed scrutiny, it is becoming increasingly clear that absolute specificity remains elusive. Nevertheless, specificity of each inhibitor class has improved with each generation of refinement. We searched the literature for a CHK1 inhibitor with high specificity and identified Gö6976 [24]. In a study where the specificities of 65 commonly used small molecule kinase inhibitors were tested for inhibition of a panel of 80 purified protein kinases, Gö6976 was shown to exhibit relative specificity against CHK1. At sub-micro molar concentration, the specificity of Gö6976 against CHK1 was over 40-fold that of CHK2, 100-fold that of MAPKAP-K2, and 30-fold that of MKK1 and MKK2 [23].

We, therefore, examined *FANCA*, *FANCG*, and *FANCD2 *mutant and paired isogenic corrected cell lines and compared the sensitivity of the lines to Gö6976 (Figure 1B). In each case, the FA pathway deficient cell line was more sensitive to Gö6976. The LC50 for the FA deficient cell lines ranged 250–500 nM whereas the LC50 for the isogenic FA proficient cell lines ranged 1–2 uM. The magnitude of the FA specific killing by Gö6976 reported here is comparable to that observed for ATM inhibitor KU55933.

While *CHK1-1 *siRNA, *CHK1-2 *siRNA, and Gö6976 each likely possess activities unrelated to CHK1 function, the recapitulation of the same phenotype using these three independent agents in multiple cell lines suggests CHK1 to be the most likely target.

### FA pathway deficient tumor cells are hypersensitive to CHK1 inhibition

To test our hypothesis that FA deficient tumor lines are hyper-dependent on CHK1 function, we tested a pair of isogenic FA proficient and deficient tumor cell lines with regard to sensitivity to CHK1 siRNA and Gö6976. The 2008 ovarian carcinoma line is deficient in FA pathway function due to methylation of the *FANCF *promoter region [6]. This cell line can be functionally corrected with an exogenously expressed *FANCF *gene to create the 2008F line. Indeed, the FA deficient 2008 cell line was found to be more sensitive to Gö6976 than the *FANCF *complemented 2008F at all doses (50 to 500 nM) tested (Figure 1C, lanes 3–8).

Since Gö6976 inhibits kinases unrelated to CHK1 [23,24], we wished to confirm our results with another inhibitor that has a relatively high specificity for CHK1 but exhibits a different specificity profile with regard to non-CHK1 kinases [23]. Such an inhibitor would unlikely recapitulate the effect of Gö6976 if the underlying mechanism was independent of CHK1. We selected UCN-01 for this purpose. Comparable to that observed for Gö6976, the FA deficient 2008 cell line was hyper-sensitive to UCN-01 relative to the FA restored 2008F cell line (Figure 1C, lanes 9–12). This finding supported our hypothesis that FA deficient tumor cells are hyper-dependent on CHK1 for cell viability.

While Gö6976 and UCN-01 exhibited differential specificity for non-CHK1 related kinases for the most part, both inhibited Protein Kinase C alpha (PRKCα) and Protein Kinase C beta 1 (PRKCβ1) in addition to CHK1 at the concentrations tested in this study [23,25]. To exclude PRKCα and PRKCβ1 inhibition as the cause underlying the FA specific tumor killing of Gö6976 and UCN-01, we tested the effect of independent siRNAs directed against PRKCα and PRKCβ1. The specificity of both siRNAs was validated in a previous study [26]. At > 80% silencing efficiency, neither siRNAs caused preferential killing of the 2008 line relative to the 2008F line. In contrast, an siRNA directed against *CHK1 *caused preferential killing of the 2008 line relative to the 2008F (Figure 1C, lanes 13–18).

While the Gö6976, UCN-01, CHK1 siRNA, PRKCα, and PRKCβ1 data sets were each individually imperfect, combined they offer strong support for the hypothesis that FA deficient tumors are hyper-dependent on CHK1 function. Overall, the FA specific tumoricidal effect of CHK1 silencing/inhibition was comparable to those that we previously reported for ATM silencing/inhibition [11].

### Knockdown of FA genes sensitizes cells to CHK1 inhibition

To demonstrate that hypersensitivity to CHK1 inhibition is the result of FA pathway deficiency rather than any specific FA genes, we next screened a number of siRNAs directed against several FA gene products for their ability to enhance the cytotoxicity of Gö6976 (Figure 1D). To this end, HeLa cells were transfected with two independent siRNAs directed against several FA genes (A, C, D1, D2, E, F, G) for 24 hrs followed by treatment by either Gö6976 or DMSO. For each siRNA, cell viability was determined as a ratio of the Gö6976 treated cells relative to the DMSO treated cells (Figure 1D). For instance, the viability of the HeLa cell treated with scrambled siRNA and Gö6976 was approximately 75% that of cells treated with scrambled siRNA and DMSO. Using this ratio, we observed that cells transfected with siRNA directed against the FA genes exhibited significantly increased sensitivity to Gö6976 relative to the scrambled sequence siRNA. Oligonucleotides targeting *BRCA1 *and *CHK1 *were also included as controls. Consistent with its role as a CHK1 inhibitor, siRNA against *CHK1 *did not further sensitize cells to Gö6976 relative to the scrambled siRNA. Similarly, knockdown of *BRCA1*, a gene required for CHK1 function in the G2/M checkpoint [27], did not further sensitize cells to Gö6976 relative to scrambled siRNA. In sum, inactivation of the FA pathway by siRNA depletion (6 FA genes tested, each gene tested with 2 independent siRNAs) consistently augmented the cytotoxicity of Gö6976.

### Synthetic lethal screen with Gö6976 revealed a predominance of FA genes

**Figure 2:**
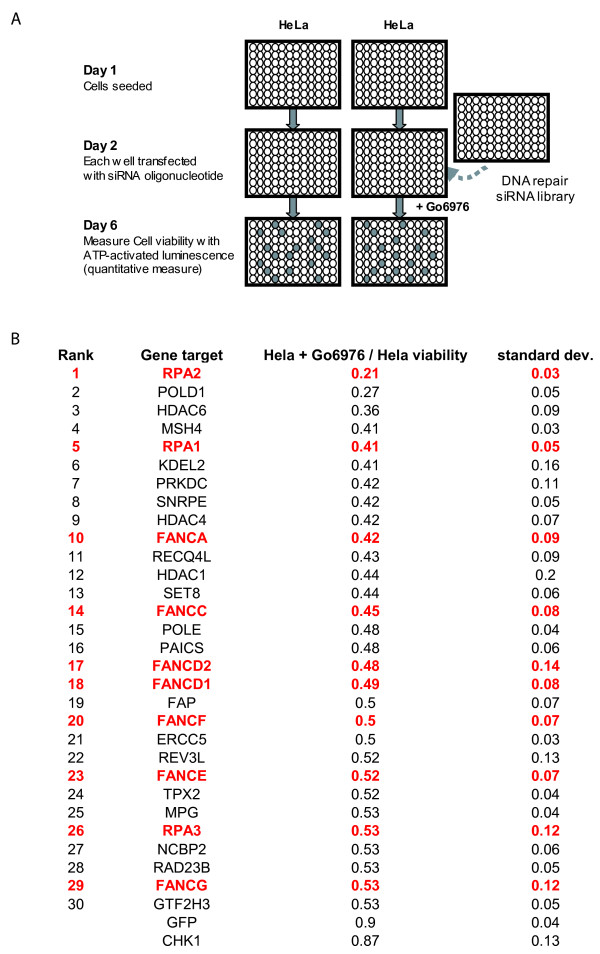
**siRNA oligonucleotide targets that are synthetically lethal with CHK1 inhibition by Gö6976**. (A) Schematic for identification of siRNA oligonucleotides that exhibited selective toxicity when combined with CHK1 inhibition by Gö6976 (i.e. synthetic lethality). Cells were plated on day 1. On day 2, each well was transfected with an siRNA oligonucleotide directed toward one DNA damage response/repair gene. On day 3, one set of cells was treated with Gö6976 (Calbiochem) at a concentration of 500 nM. The other set was treated with DMSO. On day 6, the viability of the cells in each well was measured using the Cell Titer-Glo Luminescent Cell Viability Assay kit (Promega). (B) The top 30 gene targets from the genetic screen. The top 30 targets where both independent siRNAs caused toxicity when combined with Gö6976 treatment are shown in Figure 2B. Viability and standard deviation calculation are calculated as described in Methods. Genes integral to FA pathway function are indicated in red.

Having observed the selective cytotoxicity of Gö6976 on various FA deficient cell lines, we initiated a genetic screen to identify other DNA repair defects that may predispose to such selective cytotoxicity. To this end, we screened the QIAGEN DNA repair siRNA library that consists of 460 pre-optimized siRNAs targeting 230 DNA repair/damage response genes. In this library, each gene target was represented by two distinct, pre-optimized siRNAs. We searched for genetic silencings that are selectively toxic to HeLa cells when combined with Gö6976 treatment. The screening process is outlined in Figure 2A and detailed in the Methods. In brief, each gene target is evaluated based on the average effect of the two targeting siRNAs determined in two independent screens. Using this measurement, the top 30 targets that caused selective toxicity when combined with Gö6976 treatment are shown in Figure 2B. One third of this list consisted genes required for the integrity of the FA pathway, including FANCA, FANCC, FANCD2, FANCD1, FANCF, FANCE, FANCG, RPA1, RPA2, and RPA3 [28]. The probability of such clustering by chance is less than 0.0001 (Fisher Exact Test). As such, the result of this unbiased genetic screen represents a powerful confirmation of the increased reliance of CHK1 function in FA deficient cells.

### Gö6976 induces DNA damage in FA pathway deficient cells

FA cells are characterized by DNA breakage accumulation in S phase. These breakages persist throughout S and G2 phase of the cell cycle until activation of the G2/M checkpoint. We hypothesize that *CHK1 *mediated G2/M checkpoint is required for repairing some of these DNA breaks. In this framework, CHK1 inactivation in FA deficient cells caused elevated level of DNA strand break that ultimately led to cell death.

**Figure 3:**
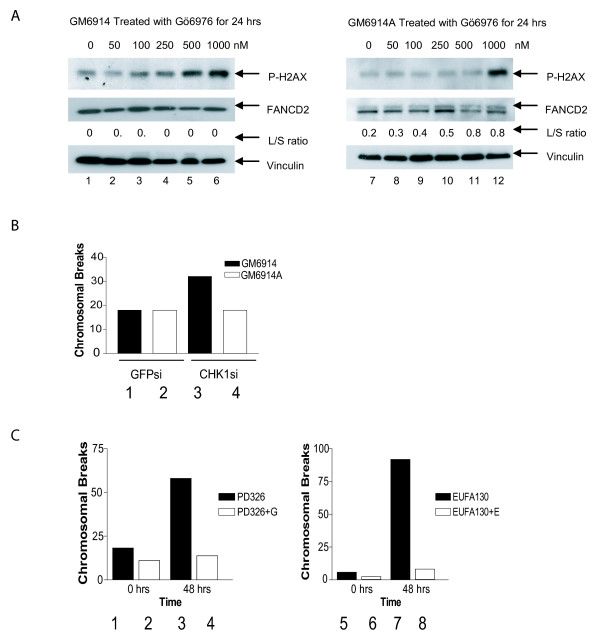
**CHK1 inhibition causes increased cell death in FA pathway deficient cells**. (A) Western blots comparing H2AX phosphorylation and FANCD2 monoubiquitination in *FANCA *mutant GM6914 cells (lanes 1 to 6) versus *FANCA *corrected GM6914+A cells (lanes 7 to 12). Each cell line was treated for 24 hrs with Gö6976 in a dose range from 0 to 1000 nM as indicated. The L/S ratio represents the ratio of the upper monoubiquitinated (Long) form of FANCD2 compared to the unmodified form (Short) as measured by densitometry. Each blot was probed for vinculin to ensure equal protein loading. (B) A graphical representation of the number of chromosomal breaks per cell as measured by metaphase spreads 72 hrs after treatment with a *GFP *targeted control siRNA (Bars 1 and 2) or a *CHK1 *targeted siRNA (Bars 3 and 4). Bars 1 and 3 represent GM6914 and bars 2 and 4 represent the isogenic corrected GM6914+A cell line. (C) A graphical representation of the number of chromosomal breaks per cell as measured by metaphase spreads 48 hrs after treatment with Gö6976 (Bars 3, 4, 7 and 8) or DMSO only (Bars 1, 2, 5 and 6). Bars 1 and 3 represent *FANCG *mutant PD326 cells and bars 2 and 4 represent isogenic corrected cells. Bars 5 and 7 represent *FANCE *mutant (EUFA130) cells and bars 6 and 8 represent isogenic EUFA130+E corrected cells. Fifty metaphase spreads were counted per experiment. Each experiment was repeated three times with similar results. Representative experiments are shown.

To test this hypothesis, we treated GM6914 (FANCA-deficient) and corrected GM6914A cells with Gö6976 for 24 hrs then assessed DNA breakage by measuring histone H2AX phosphorylation (Western blot, Figure 3A). The FA pathway deficient GM6914 (FANCA-deficient) cell line demonstrated increased H2AX phosphorylation at a concentration range of 100 nM–1 μM of inhibitor (lanes 3–6). The corrected GM6914A cell line demonstrated increased H2AX phosphorylation only at the highest concentration of Gö6976 (lane 12, 1 μM). These data indicate that the FA pathway deficient cells accumulate DNA damage at a lower level of CHK1 inhibition than the corrected line.

As an independent means of confirming the above hypothesis, we scored for metaphase spread chromosomal breaks in isogenic FA proficient and deficient cells treated with the CHK1-1 siRNA or Gö6976. Since the unrepaired strand breaks in FA cells are converted into chromosomal breaks during mitosis [29], we anticipate that *CHK1 *silencing should result in increased chromosomal breakage accumulation in FA pathway deficient cells. Indeed, the FA deficient GM6914 cell line treated with the *CHK1 *targeted siRNA demonstrated more chromosomal breakage (32 breaks per 50 cells) than the corrected line (18 breaks per 50 cells) (Figure 3B). Similarly, PD326 (*FANCD2 *deficient) and EUFA130 (*FANCE *deficient) cells demonstrated more chromosomal breakage than paired cDNA-corrected cell lines following 24 hr treatment with Gö6976 (Figure 3C). Together these data indicate that CHK1 is required to prevent the accumulation of sporadic chromosomal breaks in FA pathway deficient cells.

### Cell death after Gö6976 treatment in FA pathway deficient cells

Next we asked how Gö6976 treatment resulted in loss of viability in FA pathway deficient cells. HeLa cells were treated with siRNA targeting *FANCA *or a *GFP *control sequence. Thereafter, cells were treated with Gö6976 for 48 hrs. Cell death was assessed by flow cytometry using annexin V and propidium iodide (PI) staining.

**Figure 4:**
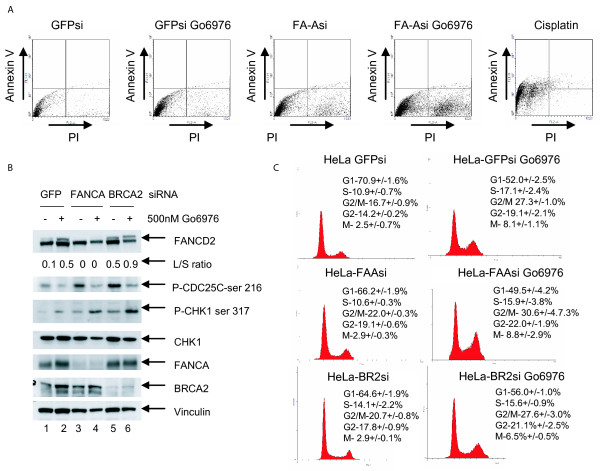
**Analysis of the mechanism of cell death and the cell cycle effect of CHK1 inhibition in FA pathway deficient cells**. (A) An annexin V/PI staining cell death assay to assess mode of cell death in HeLa cells treated with siRNA targeting *GFP*, *FANCA*, and Gö6976. Twenty-four hrs after siRNA transfection, the cells were treated with 500 nM Gö6976 or DMSO for an additional 48 hrs. Cells were then collected and subjected to annexin V/PI staining. Cells were also treated with cisplatin 10 μM for 24 hrs as a positive apoptotic control. (B) FA pathway knockdown activates CHK1 and accumulation of cells in the G2 phase of the cell cycle. Western blots measuring phosphorylation of CDC25C, CHK1, and monoubiquitination of FANCD2 in HeLa cells treated with control siRNA (lanes 1 and 2), siRNA targeting *FANCA *(lanes 3 and 4), and siRNA targeting BRCA2 (lanes 5 and 6). Cells were either treated for 24 hrs with Gö6976 (lanes 2, 4, 6) or DMSO control (lanes 1, 3, 5). Membranes were probed for vinculin to ensure equal loading. (C) Cell cycle analysis of HeLa cells treated with control GFP targeted siRNA, *FANCA *targeted siRNA or *BRCA2 *targeted siRNA followed by Gö6976 or DMSO control for 24 hrs. The mitotic population was assessed by measuring histone H3 phosphorylation by flow cytometry. Populations of cells at G1 (2N), S (between 2N and 4N), G2/M (4N), G2 (4N with no histone H3 staining), and M (4N with phosphohistone H3 staining) are given as a percentage of total cell population. Values are calculated from duplicate experiments.

Approximately 7% of the cells transfected with the *GFP *control sequence exhibited PI uptake (Figure 4A). Gö6976 treatment (500 nM) after *GFP *transfection caused a small increase in the amount of PI uptake (to 13%) relative to GFP transfection alone. About 25% of the cells exhibit PI uptake after *FANCA *depletion. Percent of cells with PI uptake was increased to 74% when combining FANCA depletion and Gö6976 treatment. This data represents another independent verification of the synthetic lethality between FA deficiency and CHK1 inactivation.

The annexin V staining was not significantly different between the various conditions tested and the positive control (cisplatin). As such, definitive conclusions regarding the mode of cell death after CHK1 inactivation could not be drawn.

### Disruption of the FA pathway activates CHK1 and results in a G2 accumulation

In view of the hypersensitivity of FA cells to Gö6976, we predicted that CHK1 may be activated in the absence of the FA pathway. To test this hypothesis, we knocked down *FANCA *or *BRCA2 (FANCD1) *in HeLa cells using siRNA and assessed CDC25C phosphorylation, as a measure of CHK1 function. In each case CHK1 was activated by knockdown of the FA gene (Figure 4B, lanes 3 and 5, anti-pCHK1 immunoblot). Addition of Gö6976 inhibited the phosphorylation of CDC25C, indicating abrogation of CHK1 function in the FA pathway deficient cells (Figure 4B, lanes 4 and 6). In addition, phosphorylation of CHK1 on serine 317 was observed following Gö6976 treatment (lanes 2, 4, 6), indicating increased ATR activity. This observation is consistent with other studies indicating an increase in ATR-mediated phosphorylation of CHK1 following inhibition of CHK1 kinase activity [30]. Also, as predicted, siRNA knockdown of FANCA or BRCA2 in HeLa cells resulted in an increase in the G2/M percentage of cells (Figure 4C), consistent with a compensatory increase in CHK1 activation of the G2/M checkpoint in these cells.

### FANCD2 knockdown sensitizes zebrafish embryos to Gö6976

**Figure 5:**
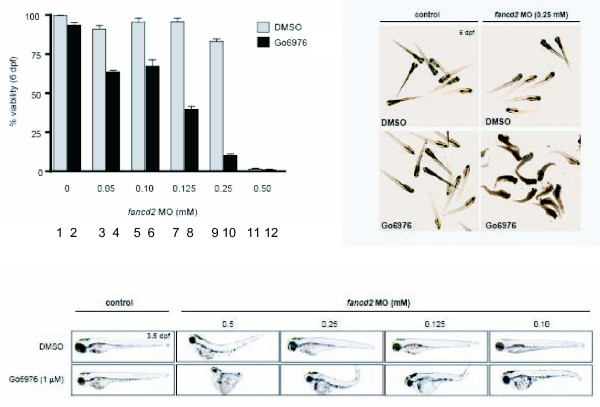
**FANCD2 depleted Zebra Fish embryos are selectively sensitive to Gö6976**. A graphical representation of zebrafish embryo viability following depletion of *FANCD2 *using an increasing concentration of a specific morpholino and treatment with Gö6976 (bars 2, 4, 6, 8, 10 and 12) or DMSO control (bars 1, 3, 5, 7, 9 and 11). Representative photographs of *FANCD2 *depleted embryos 24 hrs after Gö6976 treatment are shown. Gö6976 was used at a concentration of 0.5 uM.

To ensure that the hypersensitivity of FA deficient cells to CHK1 inhibition was not an artifact of our cell model systems, we used an *in vivo*, whole organism approach (Figure 5). We have previously described a *FANCD2 *knockdown model in zebrafish using a morpholino approach [31]. We treated zebrafish embryos with an increasing concentration of the FANCD2 morpholino after 1 uM Gö6976 treatment. The specificity of Gö6976 for CHK1 inhibition in the *in vivo *zebrafish model was previously demonstrated by our group [32]. In the absence of FANCD2 morpholino, treatment with 1 uM of Gö6976 yielded no detectable phenotype ([32] and Figure 5). However, a combined loss of the FA pathway and CHK1 function resulted in enhanced lethality of zebrafish embryos. This result confirms the synthetic lethality between FA pathway and CHK1 inactivation in an *in vivo *model.

### FA specific tumoricidal effect of CHK1 inhibition and cisplatin was synergistic

**Figure 6:**
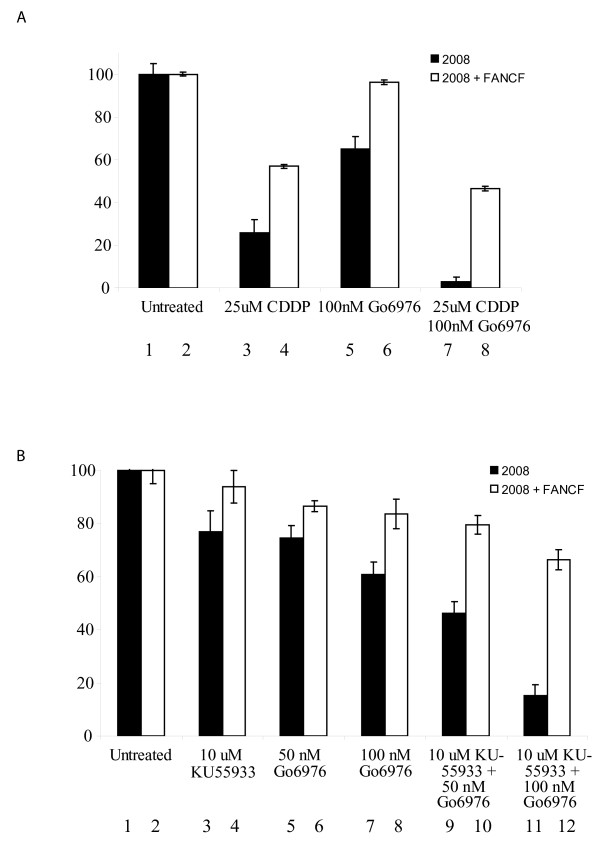
**FA specific tumoricidal activity of CHK1 inhibition in combination with Cisplatin (CDDP) treatment or ATM inhibition**. (A) Seven day viability assay comparing the response of *FANCF *deficient 2008 ovarian cancer cells (bars 1, 3, 5, 7) versus *FANCF *corrected 2008F cells (bars 2, 4, 6, 8) to 25 uM CDDP (bars 3 and 4), and 25 uM CDDP + 100 nM Gö6976 (bars 11 and 12). Bars 1 and 2 represent DMSO only controls. When fit into the Chou-Talalay mutually nonexclusive modal [33], the Combination Index (CI) is 0.7, mathematically supporting a synergistic effect. (B) 7 day viability assay comparing the response of *FANCF *deficient 2008 ovarian cancer cells (bars 1, 3, 5, 7, 9, 11) versus *FANCF *corrected 2008F cells (bars 2, 4, 6, 8, 10, 12) to 10 uM KU-55933 (bars 3 and 4), 50 nM Gö6976 (bars 5 and 6), 100 nM (bars 7 and 8), 10 uM KU-55933 + 50 nM Gö6976 (bars 9 and 10), and 10 uM KU-55933 + 50 nM Gö6976 (bars 11 and 12). Bars 1 and 2 represent DMSO only controls. When fit into the Chou-Talalay mutually nonexclusive modal [33], the Combination Index (CI) is 0.9, mathematically supporting a synergistic effect.

The selectivity of CHK1 inhibition for FA defective tumor is modest (approximately two-fold). However, CHK1 inhibition is under clinical trial in combination with cisplatin and other DNA damaging agents. Many of these DNA damaging agents, including cisplatin are also shown to have FA specific tumoricidal activities [6]. We, therefore, tested the effect of combining CHK1 inhibition with cisplatin treatment. As shown in Figure 6A, the FA deficient 2008 line was hypersensitive to cisplatin treatment and CHK1 inhibition by Gö6976. In response to either Gö6976 or cisplatin, the 2008 cells exhibited a two-fold increase in sensitivity relative to the 2008F. When subjected to a combined CHK1 inhibition and cisplatin treatment, this differential sensitivity was magnified to approximately ten-fold. When fit into the Chou-Talalay mutually nonexclusive modal [33], the Combination Index (CI) was 0.7, supporting a synergistic effect.

### Combination of ATM and CHK1 inhibition induces synergistic killing of FA deficient tumor cells

We previously demonstrated that Fanconi Anemia (FA) pathway deficient tumor cells are hypersensitive to inhibition of the Ataxia Telangiectasia Mutated (ATM) kinase (29). Having observed the synergistic FA specific effect of CHK1 inhibition and cisplatin treatment, we wished to determine whether such synergism could be achieved by combining ATM and CHK1 inhibition. To this end, we combined the lowest tumoricidal dose of the ATM inhibitor, KU-55933 (10 uM) with the lowest toxic doses of the CHK1 inhibitor, Gö6976 (50 and 100 nM). Consistent with our previous report, the 2008 cells were hypersensitive to KU-55933 (10 uM), relative to the 2008F cells (Figure 6B). Similarly, the 2008 cell line was consistently more sensitive to Gö6976 than 2008F. At the doses tested, the FA selective tumoricidal effects of ATM and CHK1 inhibition were comparable. When the two inhibitors were combined, the FA specific cytotoxicity was increased to approximately 5-fold. When fit into the Chou-Talalay mutually nonexclusive modal [33], the Combination Index (CI) was 0.9, supporting a synergistic effect.

## Discussion

We and others have previously demonstrated that epigenetic silencing of the FA pathway occurs in sporadic adult tumors [3]. It is estimated that approximately 15% of all cancers harbor defects in the FA pathway [10]. These tumors, like the FA deficient cells derived from Fanconi Anemia patients demonstrate increased accumulation of DNA strand breaks. This accumulation is attributable to defective DNA repair and DNA damage response [12,13]. As a result of these defects, compensatory repair mechanisms become activated, including the CHK1 mediated G2/M checkpoint. We hypothesize that FA deficient tumors are hyper-dependent on these pathways for viability. It follows that therapeutic gain can be achieved by selective inhibition of these compensatory pathways. We tested this paradigm by examing the effect of CHK1 inhibition in FA deficient cells.

We used four approaches to demonstrate that tumor cells deficient in the FA pathway are hypersensitive to CHK1 inhibition: 1) siRNA knockdown of FA genes 2) FA gene mutant and corrected isogenic lines; 3) a morpholino knockdown of FANCD2 in a zebrafish model; and 4) pharmacologic inhibition using two CHK1 inhibitors, Gö6976 and UCN-01. The siRNA approach most closely resembles epigenetic silencing of a normal FA gene as it occurs in a proportion of sporadic tumors. The mutant FA gene lines represent the situation in heterozygous carriers of a mutation where loss of heterozygosity results in malignancy. The zebrafish model allowed us to investigate the importance of CHK1 *in vivo*. The pharmacologic inhibition experiments are most directly translatable to clinical trials.

As with all small molecule kinase inhibitors, the specificity of Gö6976 for CHK1 is not absolute. It is well-known that additional kinases are affected by Gö6976 [23]. However, our data strongly support that the FA specific tumoricidal effect of Gö6976 is mediated through CHK1 inhibition. First, this effect of Gö6976 is recapitulated by two distinct siRNAs directed against *CHK1 *in independent cell lines (Figures 1A and 1B). Second, siRNA knockdown of *CHK1 *in a Gö6976 treated cell yield results comparable to Gö6976 treatment or *CHK1 *silencing alone (Figures 1C and 1D). Third, the effect of Gö6976 is recapitulated by another CHK1 inhibitor (UCN-01) that, for the most part, has a distinct specificity profile against non-CHK1 kinases. Finally, siRNA silencing of the two non-CHK1 targets shared by UCN-01 and Gö6976 (PRKCα and PRKCβ1) failed to cause FA specific tumor killing. While there may be inherent properties of Gö6976 that restricts its translation to clinical trial, newer classes of more specific Chk1 inhibitors are becoming available and undergoing clinical trials [34,35]. The insights derived in this study may be helpful in the design of such trials.

There are inherent limitations to CHK1 inhibition as a therapeutic strategy. Cells completely depleted for CHK1 using siRNA technology undergo severe DNA damage and die [30]. In addition CHK1 knockout mice are not viable [36]. Our data support a model in which therapeutic effect is derived from partial depletion of CHK1, rather than complete inhibition. In particular, FA pathway deficient tumor cells have a greater requirement for CHK1 function than DNA repair proficient cells. Consistent with other groups, we observed DNA breakage and toxicity at high doses of CHK1 inhibition in DNA repair competent cells [30]. FA pathway deficient cells, however, demonstrate hypersensitivity to Gö6976 at concentrations that caused little detectable phenotype in FA proficient cells (100–500 nM). These data support the existence of a therapeutic window that could be exploited in treating DNA repair deficient cancers with CHK1 inhibitors, while sparing toxicity in normal, DNA repair proficient cells.

Our results indicate that the selectivity of CHK1 inhibition for FA deficient tumor as a monotherapy is modest. However, it is one that can be exploited when combined with other modalities of treatment. For instance, we previously showed that cisplatin induced DNA lesions require activation of the FA pathway for repair. Consequently, FA deficient tumors are hypersensitive to cisplatin [6]. When CHK1 inhibition is combined with cisplatin treatment, the FA selective tumoricidal effect is increased by an order of magnitude (Figure 6A), yielding an effect that is likely clinically pertinent. As another example, we previously demonstrated that ATM and FA genes function in a compensatory manner to maintain genome integrity. The FA deficient tumor cells are, thus, hypersensitive to ATM inhibition [11]. While the selectivities of ATM inhibition and CHK1 inhibition for FA defective tumor are low individually, the effect of combining them is synergistic, yielding an effect that is likely pertinent clinically (Figure 6B).

The hypersensitivity of FA deficient cells to CHK1 and ATM inhibition suggests a framework for cancer therapy by manipulation of DNA repair. Inhibition of any one of these three pathways (FA, CHK1, and ATM) results in an increased accumulation of DNA strand breaks and chromosomal breakage that is exacerbated by the inhibition of a second pathway ([11] and Figures 1A, 3A). This inhibition translates into a modest reduction in cell survival ([11] and Figures 1A–D). Simultaneous inactivation of all three pathways (Figure 6B), however, results in a loss of cell survival that is synergistic when compared to inactivation of any two pathways ([11] and Figure 6B). These results suggest that FA, ATM, and CHK1 are functionally compensatory in the repair of DNA damage. Inactivation of any one or two pathway(s) leads to DNA damage accumulation, triggering compensatory activation of the remaining pathway(s). This compensatory activation accounts for the modest effect of CHK1 (or ATM) inhibition on FA deficient cells. Significant effect is achieved only with simultaneous inactivation of all three compensatory pathways. This framework suggests that an understanding of the network of compensatory repair pathways is a pre-requisite for meaningful manipulation of DNA repair as a therapeutic strategy.

From a biomarker perspective, our study suggests that monitoring of FA pathway activation can serve as a measure of CHK1 inhibition *in vivo*. We showed that CHK1 inhibitors or siRNAs triggered FANCD2 monoubiquitination in FA proficient cell lines (Figures 3A and 4B). Accordingly, in clinical trials, it may be useful to follow FA pathway activation as a pharmacodynamic marker of CHK1 inhibition *in vivo*. Activation of FANCD2 monoubiquitination in peripheral blood lymphocytes or tumor cells during clinical trials may allow an internal measurement of CHK1 inhibition *in vivo*. Additionally, given the hypersensitivity of FA deficient tumors to CHK1 inhibition in the context of cisplatin therapy, profiling the integrity of the FA pathway may be warranted in clinical trials involving such combination.

## Conclusion

In sum, we have identified CHK1 inhibition as a strategy for targeting FA deficient tumor cells, especially when combined with cisplatin treatment or ATM inhibition. While synergy between CHK1 inhibition and cisplatin has been documented both *in vivo *and *in vitro *previously [34], this report represents the first to demonstrate significant augmentation of this synergistic effect in FA deficient tumors. Future research should focus on the identification of DNA repair/damage response pathways absent in specific tumor types and the critical compensatory pathways activated. With such understanding, appropriate combinations of cytotoxic chemotherapy and DNA damage response/DNA repair inhibitors could be tailored for maximal therapeutic efficacy.

## Methods

### Cell Lines and Culture Conditions

The HeLa, PD326 (FANCG-deficient), GM6914 (FANCA-deficient), PD20 (FANCD2-deficient) derived cell lines have been previously described [37-39]. There were no differences between the cloning efficiency of FA proficient and deficient cells. Cells were cultured in DMEM medium (Gibco) supplemented with 15% fetal calf serum and 1 μg/ml puromycin. The EUFA130 (FANCE-deficient) and 2008 ovarian tumor line derived have also been previously described [6,40] and were cultured in RPMI medium (Gibco) supplemented with 15% fetal calf serum and 1 μg/ml puromycin. Cisplatin (Sigma), UCN-01 (Sigma), and Gö6976 (Calbiochem) were dissolved in DMSO at 1 mg/ml and used at the specified concentrations. KU-55933 (Sigma) was prepared as 10 mmol/L stock solution in DMSO.

### siRNA Library screen

The QIAGEN DNA damage response DNA repair siRNA library targeting 230 DNA damage response genes was purchased in seven 96-well plates. Each plate also contained 2 GFP-targeted siRNAs, 2 LacZ-targeted siRNAs, and 16 wells containing no siRNA as controls. HeLa cells were seeded in 2 sets of 7 black 96 well plates (BD biosciences) at a count of 1000 cells per well in 80 ul of DMEM medium with 15% FCS. Cells from each set were individually transfected with siRNA from the Qiagen DNA repair library as previously described [11]. After 72 hrs one set of cells was treated with Gö6976 (Calbiochem) at a concentration of 500 nM. The other set was treated with DMSO alone. Forty-eight hrs later, the viability of the cells in each well was measured using the Cell Titer-Glo Luminescent Cell Viability Assay kit (Promega). The experiment was performed twice to allow statistical analysis of the targets. The corrected viability for each siRNA oligonucleotide was calculated as a percentage of the mean viability of the 16 non-siRNA treatment control wells on each plate. The corrected viability of the Gö6976 treated cell line was divided by the corrected viability of the DMSO control treated cell line for each gene target to calculate the relative viability for each respective gene target. The mean relative viability between the Gö6976 and DMSO treated cell line for each gene target, along with the standard error of the mean (SEM), was calculated from four individual corrected viability values that represent duplicate results from the two different oligonucleotides on each plate targeting a particular gene.

### Zebrafish data

1-cell stage wild-type zebrafish embryos (*AB *strain) were injected with *fancd2 *MO [31]. At 24 hrs post-fertilization (hpf), embryos were dechorionated and incubated in standard embryo medium containing 1% DMSO and 1 μM Gö6976 (or DMSO as control). Viability was assayed at 6 dpf in three independent experiments. Forty-eight hpf and 6 dpf embryos were photographed with a Nikon Digital Sight DS-U1 camera mounted on a Nikon SMZ1500 microscope.

### Cell Viability Assay

All viability experiments were done in triplicates and repeated three times. For the Gö6976 and the drug combination studies, cells were seeded in 96-well plates at a density of 250–500 cells per well on day 1. The cells were treated with various combinations of 10 uM KU-55933 (Sigma-Aldrich), 50 nM Gö6976 (Calbiochem), 100 nM Gö6976, 25 uM cisplatin (Sigma-Aldrich), or DMSO (negative control) on day 2. Seven days after treatment, cellular viability was measured using the Cell Titer-Glo Luminescent Cell Viability Assay kit (Promega). The mean cellular viability and standard error measurement were calculated as a percentage of the untreated controls from three separate experiments.

For viability assays following *CHK1 *or control *GFP *targeted siRNA transfection, cellular viability was measured using the Cell Titer-Glo Luminescent Cell Viability Assay kit (Promega) 7 days after transfection. Cellular viability for each well was calculated as a percentage of the mean viability of *GFP *targeted siRNA treated cells. The mean cellular viability and standard error of the mean was calculated and plotted using GraphPad Prism version 3 (GraphPad Software, San Diego California USA). Each viability experiment was repeated at least three times.

### Cell Cycle analysis and P-H3 staining

Cell cycle profiles were measured by flow cytometry as previously described [41]. For p-H3 measurement, cells were washed with phosphate-buffered saline (PBS), and fixed in 70% ethanol for 24 hrs. Cells were then permeabilized in 1 ml of 0.25% Triton X-100 in PBS, and incubated on ice for 15 min. After centrifugation, the cell pellet was resuspended in 100 μl of PBS containing 1% bovine serum albumin (BSA) and phosphohistone H3 polyclonal antibody (1:100) (Upstate Biotechnology, Lake Placid, N.Y.) and incubated for 3 hrs at room temperature. The cells were then washed with PBS containing 1% BSA and incubated with fluorescein isothiocyanate-conjugated goat anti-rabbit immunoglobulin G antibody (Jackson ImmunoResearch Laboratories, Inc., West Grove, Pa.) diluted at a ratio of 1:30 in PBS containing 1% BSA. After 30 mins the cells were washed in PBS and resuspended in 25 μg of propidium iodide (PI) (Sigma, St. Louis, Mo.)/ml and 0.1 mg of RNase A (Sigma)/ml in PBS for 30 minutes at room temperature. The samples were analyzed using a Becton Dickinson (San Jose, Calif.) FACSCalibur flow cytometer/cell sorter.

### Cell Death Assay

Cells were seeded at 50% confluence in 100 mm dishes and treated for 48 hrs with 500 nM Gö6976 or 8 μl DMSO control. Cell death was measured using the Annexin V-FITC Apoptosis Detection Kit I (BD Pharmingen).

### Clonogenic Assays

All clonogenic experiments were done in triplicate and repeated three times. The 2008 and 2008F cell lines were each seeded at a density of 500 cells in a 100 mm diameter dish and after 6 hrs treated with Gö6976 at 50, 100 or 500 nM for 24 hrs or DMSO. After 10 days, monolayers were washed once with PBS and fixed for 10 min at room temperature in 10% (vol/vol) methanol and 10% (vol/vol) acetic acid. Adherent colonies were stained for 5 to 10 min at room temperature with 1% (wt/vol) crystal violet (Sigma) in methanol. The mean colony count and standard error of the mean were calculated.

### siRNA Oligonucleotides

The following siRNA target sequences were used: *GFP *(5'-AACACTTGTCACTACTTTCTC-3'), *CHK1-1 *(5'-AAGAAGCAGTCGCAGTGAAGA-3'), *CHK1-2 *(5'-CCACCTCATCATAACAACAAT-3'), *BRCA2 *(Dharmacon, Smartpool reagent M-003462-00-0005) and *FANCA *(Santa Cruz sc-40567). Other siRNAs used were taken from the QIAGEN DNA damage response DNA repair or the kinome libraries. These sequences will be available upon request. For all experiments, greater than 80% knockdown was achieved with each siRNA as gauged by qPCR.

### siRNA oligonucleotide verification

Cell lines were transfected with 20 nM of siRNA oligonucleotides using HiPerfect transfection reagent (QIAGEN, Valencia, CA) following the manufacturer's instructions. The QIAGEN QuantiTect SYBR Green RT-PCT Kit was used to quantify the efficiency of gene silencing per manufacturer's instructions. The expression of each gene was normalized using the housekeeping gene GAPDH. Knockdown for each siRNA was compared to a scrambled siRNA control. Data were analyzed with a BioRad iCycler.

### Immunoblotting

Cells were lysed with 1× sample buffer (50 mM Tris-HCl (pH 6.8), 0.6% 2-mercaptoethanol, and 2% sodium dodecyl sulfate (SDS)) and boiled for 20 min. The lysate was added to 1× loading buffer (50 mM Tris-HCl (pH 6.8), 0.6% 2-mercaptoethanol, 2%SDS, 20% glycerol) and subjected to polyacrylamide SDS gel electrophoresis. For FANCA, FANCF, FANCD2 or vinculin, 10 μg of protein was run on a 3–8% tris-acetate gradient gel (Invitrogen). For the detection of all other proteins 10 μg was run on a 4–12% bis-tris gel (Invitrogen). Following electrophoresis, proteins were transferred to a PVDF membrane using a submerged transfer apparatus (Bio-Rad). Membranes were then probed with the following antibodies: anti-FANCD2 (1:1000; Santa Cruz Cat#sc-20022), anti-FANCA (1:1000; [42]) anti-FANCF (1:1000, [43], anti-BRCA2 (1:1000, Calbiochem Cat#OP95), anti-phosphoH2AX Ser 139 (1:2000; Upstate Cell Signaling Solutions Cat#07-164), anti-phopsho-317-CHK1 (1:500; Cell Signaling Cat#2344), anti-CHK1(G-4) (Santa Cruz Cat#8480), antiphospho-CDC25C (1:1000; Cell Signaling Cat#4901), anti-GAPDH (1:3000; Abcam Cat#ab9484), and anti-vinculin (1:2000; Santa Cruz Cat#sc25336).

### Cytogenetic Analysis

Chromosomal breakage was analyzed on metaphase spreads as previously described [44]. Fifty spreads were scored for each experiment, and each experiment was repeated twice. PD326, GM6914 and EUFA130 cells and isogenic corrected cells were treated with Gö6976 500 nM for 48 hrs prior to analysis. In addition, GM6914 and isogenic corrected cells were treated with *GFP *siRNA or *CHK1 *targeted siRNA for 72 hrs prior to analysis.

## Abbreviations

FA: Fanconi Anemia.

## Competing interests

This work was supported by a Susan G. Komen Breast Cancer Foundation Fellowship (R.D. K.), National Institutes of Health Grants RO1HL52725, RO1DK43889, PO1150654, P50CA105009-01, PO1HL54785 (A.D.), Centers for Medical Counter Measures Against Radiation (U19A1067751), a grant from the Leukemia/Lymphoma Society LLS2007 (A.D.), and the Burroughs Wellcome Fund Career Awards for Medical Sciences (C.C.).

## Authors' contributions

CCC conceived of the study, performed the siRNA screen, conducted the siRNA validation experiments, and completed the manuscript. SS and ATL conducted the zebrafish experiments. RDK performed all other experiments. AD supervised the design of the experiments and finalized the manuscript. All authors read and approved the final manuscript.

## Authors' information

CCC was a former Damon Runyon Post-doctoral fellow and is a current Burroughs Wellcome Fund investigator. RDK was a former Susan G. Komen Breast Cancer Foundation Fellow. ATL is the current vice chair for research at the Dana Farber Cancer Institute. AD is the chief of the division of genome stability at the Dana Farber Cancer Institute.
